# Aneurisma Perfurado da Válvula Mitral: Um Problema de Inflamação ou Gradientes de Pressão?

**DOI:** 10.36660/abc.20211031

**Published:** 2022-09-30

**Authors:** Inês Oliveira, Isabel Cruz, Ana Neto, Bruno Bragança, Glória Abreu, João Azevedo, Aurora Andrade

**Affiliations:** 1 Departamento de Cardiologia Centro Hospitalar Tâmega e Sousa Penafiel Portugal Departamento de Cardiologia , Centro Hospitalar Tâmega e Sousa, Penafiel – Portugal

**Keywords:** Endocardite Bacteriana, Cardiomiopatia Hipertrófica, Válvula Mitral/anormalidades, Inflamação, Aneurisma Valvular, Diabetes Mellitus/complicações, Dislipidemia/complicações, Diagnóstico por Imagem

## Relato de caso

Aneurismas da válvula mitral (AVM) são incomuns e geralmente evoluem de forma aguda como manifestação de endocardite infecciosa (EI). ^1^ O AVM como uma complicação tardia de EI em pacientes com cardiomiopatia hipertrófica obstrutiva (CMH) é bastante incomum, levantando considerações em relação ao papel do processo infeccioso e das condições hemodinâmicas inerentes à cardiomiopatia. ^1 , 2^ Apresentamos um relato de caso de um paciente com CMH e ruptura de aneurisma no folheto da válvula mitral (VM) secundário a EI tratada anteriormente.

**Figura 1:**
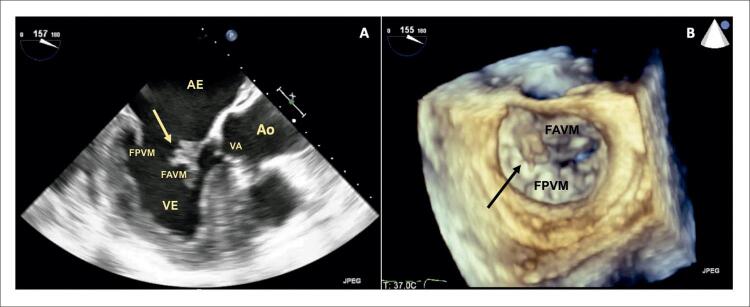
A) Avaliação ecocardiográfica transesofágica bidimensional revelando hipertrofia do ventrículo esquerdo e movimento sistólico anterior da válvula mitral, como massa polipoide móvel no lado atrial do folheto anterior da válvula altamente sugestivo de vegetação (→); B) Avaliação ecocardiográfica transesofágica tridimensional, vista frontal da válvula mitral revelando uma estrutura polipoide aderente a seu folheto anterior (→), correspondendo a uma vegetação. FAVM: folheto anterior da válvula mitral; Ao: aorta; VA: válvula aórtica; AE: átrio esquerdo; VE: ventrículo esquerdo; FPVM: folheto posterior da válvula mitral.

**Figura 2:**
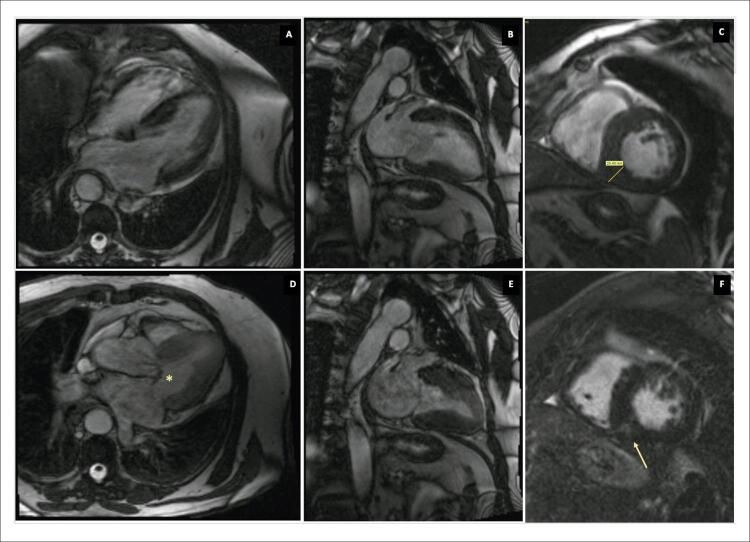
A, B, C, E) Avaliação de imagens por ressonância magnética mostrando hipertrofia assimétrica do ventrículo esquerdo, com espessura máxima de 20 mm na parede inferoseptal (C)); D) vista de três câmaras mostrando movimento sistólico anterior do folheto da válvula mitral (*); F) Vista do eixo curto médio-ventricular revelando a presença de realce tardio pelo gadolínio localizado na parede média inferoseptal (→), sugerindo fibrose miocárdica

**Figura 3:**
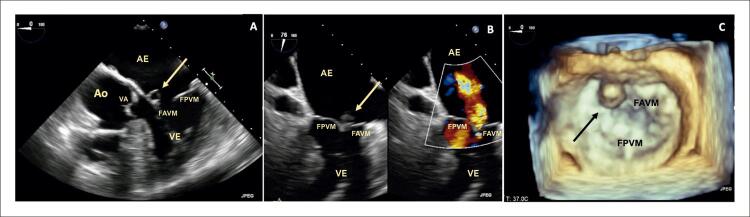
A) Avaliação ecocardiográfica transesofágica bidimensional revelando uma protuberância sacular no folheto anterior da válvula mitral com expansão sistólica na direção do átrio esquerdo, sugerindo um aneurisma no folheto (→); B) Regurgitação mitral moderada (grau II/IV) através do aneurisma do folheto anterior da válvula mitral; C) Avaliação ecocardiográfica transesofágica tridimensional, vista frontal mostrando a protuberância sacular localizada no folheto anterior da válvula mitral correspondendo ao aneurisma do folheto (→). FAVM: folheto anterior da válvula mitral; Ao: aorta; VA: válvula aórtica; AE: átrio esquerdo; VE: ventrículo esquerdo; FPVM: folheto posterior da válvula mitral.

Um paciente do sexo masculino de 68 anos de idade com diabetes mellitus tipo 2 e dislipidemia foi internado com um histórico de 3 semanas de mal-estar, febre e dor abdominal recente no lado esquerdo. O exame físico revelou um sopro cardíaco sistólico de grau II/VI no ápex cardíaco, febre, sensibilidade abdominal no quadrante superior esquerdo e lesões purpúricas nos membros inferiores. O exame de sangue indicou neutrofilia, proteína C reativa de 211 mg/L, além de culturas sanguíneas positivas para Staphylococcus aureus sensível à meticilina. A tomografia computadorizada abdominal revelou embolização do baço, sem abcessos.Os ecocardiogramas transtorácico (ETT) e transesofágico (ETE) revelaram uma massa polipoide altamente móvel e no lado atrial do folheto anterior da VM sugestiva de vegetação, com regurgitação mitral leve sem evidência de abscesso, aneurisma ou perfuração da válvula; hipertrofia assimétrica do ventrículo esquerdo (VE) sem presença de gradiente sistólico intraventricular (GSIV) e movimento sistólico anterior (MSA) da VM ( Figura 1 ). O diagnóstico de EI e CMH foi estabelecido, sendo tratado adequadamente com flucloxacilina com evolução clínica favorável. Depois de três meses de acompanhamento, foi realizada ressonância magnética cardíaca, confirmando o diagnóstico de CMH: aumento da massa do VE (96 g/m ^2^ ) com hipertrofia do ventrículo esquerdo (espessura máxima de 20 mm na parede inferoseptal), sem defeitos de perfusão, mas com evidência de realce tardio pelo gadolínio intramural na parede inferoseptal, e MSA da VM ( figura 2 ). O ecocardiograma foi repetido e, além de evidência de CMH obstrutiva com um GSIV em repouso de 44 mmHg, foi identificado um aneurisma do folheto anterior da VM. Foram observados dois jatos mitrais regurgitantes, um devido à coaptação incompleta dos folhetos e outro devido ao aneurisma perfurado, quantificando a regurgitação mitral (RM) global em moderada (grau II/IV) ( figura 3 ). A dose de betabloqueador foi aumentada e adotou-se a estratégia de acompanhamento próximo. Verificou-se a manutenção das características do aneurisma no acompanhamento ambulatorial do paciente após 2 anos.

O AVM é uma doença rara, mas potencialmente séria. A literatura publicada mostra que ela se desenvolve principalmente no folheto anterior da VM na manifestação aguda da EI da válvula aórtica (VA), devido à direção do jato aórtico regurgitante e disseminação secundária do processo infeccioso até a VM. ^1 , 3^ Isso leva a inflamação localizada, fraqueza de tecidos, formação de abscesso com drenagem posterior e, eventualmente à formação de aneurisma. ^1 , 3^ As características ecocardiográficas variam de uma protuberância sacular, geralmente difícil de identificar devido à presença de vegetações, a uma grande protuberância do folheto na direção do átrio esquerdo, que pode estar associada a vários graus de RM e formação de trombos. ^1 , 3^ As manifestações clínicas e a indicação cirúrgica dependem do significado hemodinâmico das lesões valvulares. ^1 , 3^ Num manuscrito de Reid et al., ^1^ cinco pacientes com AVM do folheto anterior da VM (como manifestação da EI da VA) são descritos considerando as características clínicas, ecocardiográficas e patológicas. Foram relatados sintomas de insuficiência cardíaca e vários graus de regurgitação valvular. Quatro passaram pela substituição de VA e apenas dois tiveram intervenção da VM, já que os AVM não haviam sido diagnosticados – foram identificados na autópsia, destacando a importância de uma avaliação pré-operatória detalhada. ^1^ Os estudos de autópsia detectaram que o AVM tinha material necrótico cercado por vegetações no folheto anterior, poupando-se os folhetos posteriores da VM. ^1^

Menos frequente ainda é o achado de AVM em pacientes como doenças do tecido conjuntivo, que raramente foram relatados, sugerindo uma conexão entre fragilidade do tecido e seu desenvolvimento. ^3^

O folheto anterior da VM também é o folheto da válvula mais frequentemente afetado na EI em pacientes com CMH. ^2 , 4^ Embora atualmente não exista associação direta estabelecida entre EI e CMH, essa cardiomiopatia era considerada uma condição de risco moderado para o desenvolvimento do EI, tendo em vista os relatórios publicados mostrando uma associação entre a doença infecciosa e CMH. ^2^ O aumento da suscetibilidade à EI parece se dever principalmente a anormalidades estruturais nos folhetos da VM, tais como o alongamento do folheto, deslocamento dos músculos papilares e microtrauma contínuo do endocárdio valvular pelo contato mitral-septal durante MSA. ^2 , 4 , 5^ Também foi sugerido que a obstrução do canal de saída do VE é um fator contribuinte importante no desenvolvimento de MSA e EI, já que a grande diferença de pressão e o estresse de cisalhamento na VM nessa manifestação leva a MSA, e a alta velocidade e o fluxo sanguíneo turbulento na CMH obstrutiva lesiona o endocárdio da válvula. ^2 , 4 , 6^

Neste caso, o AVM se desenvolveu como complicação tardia da EI da própria VM, que é incomum, não apenas por ser encontrado com mais frequência na EI de VA, mas também devido à sua apresentação subaguda. Acreditamos que características estruturais do folheto anterior da VM típicas de CMH formaram um substrato suscetível à formação de vegetações nesse folheto. Especulamos que a inflamação após a conclusão da terapia com antibiótico e a resolução dos marcadores inflamatórios sistêmicos, além da presença de MSA da VM e alto GSIV, tiveram um papel na formação de aneurisma no folheto anterior da VM.

Dependendo das condições hemodinâmicas e do ambiente local, o tamanho do aneurisma pode aumentar e complicar, levando à deterioração clínica. ^5 , 7^ A complicação mais nefasta é a RM grave aguda com edema pulmonar, devido ao rompimento do AVM, ou como resultado do defeito de coaptação do folheto causada por seu efeito de massa. ^1 , 3^ Na manifestação da CMH obstrutiva, a presença de um GSIV significativo parece contribuir para o crescimento do aneurisma, aumentando sua propensão de gerar uma protuberância na direção do átrio, crescer e se perfurar. ^7^ Em nosso caso, o tamanho do AVM permaneceu razoavelmente estável ao longo do tempo. Nossa hipótese é de que o aumento na dose de betabloqueador impediu o crescimento do AVM, diminuindo o GSIV característico dessa cardiomiopatia e a turbulência do fluxo contra o folheto da VM - uma hipótese que ainda requer estudo confirmatórios, já que faltam dados publicados sobre a questão.

Em relação ao diagnóstico, o ETT e o ETE são os métodos de escolha para a identificação de AVM e de perfuração dos folhetos da válvula. ^8 , 9^ O ETE tem mais sensibilidade e especificidade para lesões aneurismáticas, permitindo uma caracterização morfológica mais precisa do tecido. ^9^ A tomografia computadorizada e as imagens por ressonância magnética são úteis na avaliação valvular, mas há poucos dados que consideram sua função na avaliação e no diagnóstico dos aneurismas valvulares. ^10 , 11^

A abordagem ideal ao AVM não foi definida, dependendo do tamanho e das consequências hemodinâmicas da lesão valvular. Em pequenos aneurismas com RM leve ou moderada, uma abordagem conservadora parece razoável, conforme decidido neste caso; porém, na RM grave, a cirurgia é a única opção razoável. ^3 - 7^

Em conclusão, o AVM é uma complicação rara, mas potencialmente fatal, da EI. O objetivo deste caso é destacar possíveis complicações desse processo infeccioso e lembrar que certas doenças cardíacas podem ter maior propensão a desenvolver complicações e resultados desfavoráveis, devido à interação entre inflamação e gradientes de pressão.
