# Supplementary material for: Aneurisma Perfurado da Válvula Mitral: Um Problema de Inflamação ou Gradientes de Pressão?
Source: Arq Bras Cardiol. 2022 Sep 30;119(5):809–12. [Article in Portuguese] doi: 10.36660/abc.20211031 (PMC9750205; doi:10.36660/abc.20211031)
Supplement: Translation

# Translation: Mitral Valve Perforated Aneurysm: An Issue of Inflammation or Pressure Gradients?

**Authors:** Inês Oliveira, Isabel Cruz, Ana Neto, Bruno Bragança, Glória Abreu, João Azevedo, Aurora Andrade

**Affiliations:** 1 Portugal Departamento de Cardiologia, Centro Hospitalar Tâmega e Sousa, Penafiel – Portugal

**Keywords:** Endocarditis, Bacterial, Cardiomyopathy, Hypertrophic/complications, Mitral Valve/abnormalities, Inflammation, Aneurysm, Valvular, Diabetes Mellitus/complications, Dyslipidemia/complications, Diagnostic, Imaging

## Case Report

Mitral valve aneurysms (MVA) are uncommon and usually develop acutely in the setting of infective endocarditis (IE).^1^ MVA as a late complication of IE in a patient with obstructive hypertrophic cardiomyopathy (HCM) is rather unusual, raising considerations regarding the role of the infectious process and the hemodynamic conditions inherent to the cardiomyopathy.^1 , 2^ We present a case report of a patient with HCM and a ruptured aneurysm of the mitral valve (MV) leaflet, secondary to previously treated IE.

A 68-year-old male patient with type 2 diabetes mellitus and dyslipidemia was admitted to a hospital with a 3-week history of malaise, fever, and recent left-sided abdominal pain. His physical examination revealed a grade II/VI systolic heart murmur at the cardiac apex, fever, abdominal tenderness in the left upper quadrant, and purpuric lesions in the inferior limbs. Blood analyses showed neutrophilia and C-Reactive Protein of 211mg/L. Positive blood cultures indicated Staphylococcus aureus methicillin-sensitive. Abdominal Computed Tomography revealed spleen embolization, with no abscess. Transthoracic (TTE) and transesophageal echocardiography (TEE) disclosed a highly mobile polypoid mass in the atrial side of the anterior MV leaflet, suggestive of a vegetation, with mild mitral regurgitation and no evidence of abscess, aneurysm, or valve perforation; asymmetrical left ventricular (LV) hypertrophy with the presence of an increased intraventricular systolic gradient (IVSG) and systolic anterior motion (SAM) of the MV ( Figure 1 ). The diagnosis of IE in HCM was established and treated accordingly with flucloxacillin, presenting a favorable clinical evolution. After three months of follow-up, cardiac magnetic resonance was performed, confirming the diagnosis of HCM: an increased LV mass (96g/m^2^ ) with asymmetrical left ventricular hypertrophy (maximal thickness of 20mm in the inferoseptal wall), with no perfusion defects but with evidence of intramural late gadolinium enhancement in the inferoseptal wall,and SAM of the MV ( Figure 2 ). Echocardiography was repeated and, in addition to evidence of obstructive HCM with a rest IVSG of 44 mmHg, an aneurysm of the anterior leaflet of the MV was identified. Two mitral regurgitant jets were observed, one due to incomplete coaptation of the leaflets and another through the perforated aneurysm, quantifying global mitral regurgitation (MR) in a moderate (grade II/IV) ( figure 3 ). The beta-blocker dose was increased, and a strategy of close follow-up was adopted. Maintenance of the characteristics of the aneurysm was verified in outpatient follow-up after 2 years.

**Figure 1:** A) 2-D Transesophageal echocardiographic evaluation revealing left ventricular hypertrophy and systolic anterior motion of the mitral valve, with a polypoid mobile mass in the atrial side of the anterior valve leaflet, highly suggestive of a vegetation (→); B) 3-D Transesophageal echocardiographic evaluation, “en-face” view of the mitral valve, revealing a polypoid structure adherent to its anterior leaflet (→), corresponding to a vegetation. AMVL: anterior mitral valve leaflet; Ao: aorta; AV: aortic valve; LA: left atrium; LV: left ventricle; MV: mitral valve; PMVL: posterior mitral valve leaflet.

**Figure 2:** A, B, C, E) Magnetic Resonance Imaging evaluation showing asymmetrical left ventricular hypertrophy, with maximal thickness of 20 mm in the inferoseptal wall (C)); D) 3-Chamber view depicting systolic anterior motion of the mitral valve leaflet (*); F) Mid-ventricular short-axis view revealing the presence of intramyocardial late gadolinium enhancement located in the inferoseptal mid wall (→), suggestive of myocardial fibrosis.

**Figure 3:** A) 2-D Transesophageal echocardiographic evaluation depicting a saccular bulge in the anterior mitral valve leaflet with systolic expansion towards the left atrium, suggestive of a leaflet aneurysm (→); B) Moderate mitral regurgitation (grade II/IV) through the aneurysm of the anterior mitral valve leaflet; C) 3-D Transesophageal echocardiographic evaluation, “en-face” view showing the saccular bulge located on the anterior mitral valve leaflet corresponding to the leaflet aneurysm (→). AMVL: anterior mitral valve leaflet; Ao: aorta; AV: aortic valve: LA: left atrium: LV: left ventricle: MV: mitral valve; PMVL: posterior mitral valve leaflet.

MVA are rare but potentially serious conditions. Published literature shows that they mostly develop in the anterior MV leaflet in the acute setting of IE of the aortic valve (AV), due to the aortic regurgitant jet direction and secondary spreading of the infectious process to the MV.^1 , 3^ This leads to localized inflammation, tissue weakness, abscess formation with posterior drainage, and eventually to the formation of an aneurysm.^1 , 3^ Echocardiographic features vary from a small saccular bulges, often difficult to identify due to the presence of vegetations, to a large leaflet protuberance towards the left atrium, which may be associated with various degrees of MR and thrombus formation.^1 , 3^ Clinical manifestations and surgical indications depend on the hemodynamic significance of the valvular lesions.^1 , 3^ In a manuscript from Reid et al.,^1^ five patients with MVA of the anterior MV leaflet, in the setting of AV IE, were described, considering clinical, echocardiographic, and pathological features. Symptoms of heart failure and various degrees of valve regurgitation were reported. Four underwent AV replacement, while only two received MV intervention, as the MVA had not been previously diagnosed – these were identified in an autopsy, highlighting the importance of a detailed preoperative evaluation.^1^ Autopsy studies found that MVA had necrotic material surrounded by vegetations in the anterior leaflet, with a sparring of the posterior MV leaflets.^1^

Even less frequent is the finding of MVA in patients with connective tissue diseases, which has seldom been reported, suggesting a connection between tissue fragility and their development.^3^

The anterior MV leaflet is also the most often affected valve leaflet in IE in patients with HCM.^2 , 4^ Although nowadays no direct association is established between IE and HCM, this cardiomyopathy used to be considered a moderate-risk condition for IE development, in view of the published reports depicting an association of the infectious disease and HCM.^2^ Increased leaflet susceptibility to IE seems to be primarily due to structural abnormalities in MV leaflets, such as leaflet elongation and papillary muscle displacement, along with continuous microtrauma of the valve endocardium by mitral-septal contact during SAM.^2 , 4 , 5^ LV outflow tract obstruction has also been suggested as an important contributor for SAM and IE development, as the high pressure difference and the shear stress on the MV in this setting leads to SAM, and the high velocity and turbulent blood flow in obstructive HCM damages the valve endocardium.^2 , 4 , 6^

In this case, the MVA developed as a late complication of IE of the MV itself, which is unusual, not only because it is more often found in AV IE, but also due to its subacute presentation. We believe that structural characteristics of the anterior MV leaflet, typical of HCM, formed a susceptible substrate for vegetations to develop in this leaflet. It can therefore be speculated that possible persistent latent inflammation after completing antibiotic therapy and resolving systemic inflammatory markers, coupled with the presence of the SAM of the MV and a high IVSG, have played a role in aneurysm formation in the anterior MV leaflet.

Depending on hemodynamic conditions and local environment, aneurysm size may increase and complicate, leading to clinical deterioration.^5 , 7^ The most dreadful complication is acute severe MR and pulmonary edema due to MVA perforation or as a result of a leaflet coaptation defect caused by its mass effect.^1 , 3^ In the setting of obstructive HCM, the presence of a significant IVSG seems to be a contributor to the growth of an aneurysm, increasing its propensity to bulge towards the atrium, growing and perforating.^7^ In our case, MVA size has remained reasonably stable over time. We thus hypothesize that the increase in beta blocker dose may have prevented MVA growth, decreasing the IVSG characteristic of this cardiomyopathy and the flow turbulence against the MV leaflet – a hypothesis that still requires further confirmatory studies, as data published on the issue is scarce.

Regarding the diagnosis, TTE and TEE are the methods of choice for identifying MVA and perforation of the valve leaflets.^8 , 9^ TEE has a higher sensitivity and specificity for aneurysmal lesions, enabling a more accurate morphologic tissue characterization.^9^ Computed tomography and magnetic resonance imaging are useful in valvular evaluation, but little data exists considering their role in the evaluation and diagnosis of valvular aneurysms.^10 , 11^

An optimal approach to MVA has not yetbeen defined, depending on the size and hemodynamic consequences of the valvular lesion. In small aneurysms with mild or moderate MR, a conservative approach seems reasonable, as decided in this case, while in severe MR, surgery is the only reasonable option.^3 - 7^

In conclusion, MVA are rare but potentially life-threatening complications of IE. The purpose of this case is to highlight possible complications of this infectious process and to remind that certain heart conditions may have an increased propensity to develop complications and unfavorable outcomes due to the interplay between inflammation and pressure gradients.
